# Books and Magazines

**Published:** 1904-10

**Authors:** 


THE AMERICAN ILLUSTRATED MEDICAL DICTIONARY.—For practitioners and students. A complete dictionary of the terms used in medicine, surgery, dentistry, pharmacy, chemistry, and the kindred branches, including much collateral information of an encyclopediac character, together with new and elaborate tables of arteries, muscles, nerves, veins, etc.; of bacilli, bacteria, micrococci, streptococci; eponymic tables of diseases, operations, signs and symptoms, stains, tests, methods of treatment, etc., etc. By W. A. Newman Dorland, A. M., M. D., editor of the "American Pocket Medical Dictionary." Third edition, thoroughly revised; handsome large octavo, nearly 800 pages, bound in full flexible leather. Philadelphia, New York, London: W. B. Saunders & Company. 1903. Price, $4.50 net; with thumb index, $5 net.

The American Illustrated Medical Dictionary certainly fulfills all claims of its author, and with its first edition, he endeavors to present, in a single volume of convenient size, an up-to-date medical dictionary, sufficiently full for the varied requirements of all classes of medical men. The author has succeeded admirably in giving the profession all that he claims for his book. The definitions are well worded, clear and concise, and are kept within the bounds of brevity. A large amount of information is arranged in a tabulated form for the convenience of the student and busy practitioner. The important features have received most careful attention, and the book is well illustrated, and is especially appropriate for making plain its text. This is the third edition of the work, which goes to show the large patronage given it, and its author desires to keep it strictly up-to-date by including in it all of the newly coined words, and any additional information which has been added to medicine during the past year. The work certainly can be looked upon as one of the standard, practical dictionaries, in a handy size, covering everything in the field of medicine, including surgery, dentistry, pharmacy, chemistry and kindred branches. It is an admirable testimony of the book-maker's art, and Messrs. Saunders & Co. are to be congratulated.—*Texas Medical News.*

---

A TEXT-BOOK OF DISEASES OF WOMEN. By Charles B. Penrose, M. D., Ph. D., formerly Professor of Gynecology in the University of Pennsylvania. Fifth edition, thoroughly revised. Octavo volume of 539 pages, with 221 fine original illustrations. Philadelphia, New York, London: W. B. Saunders & Company. 1904. Cloth, $3.75 net.

With astonishing regularity, a new edition of this excellent textbook is called for, and it appears to be in as great favor with physicians as with students. Indeed, this book has taken its place as the ideal work for the general practitioner. The author presents the best teaching of modern gynecology, untrammeled by antiquated ideas and methods. In most instances only one plan of treatment is described.

The new edition has been carefully revised, much new matter has been added, and a number of new original illustrations have been introduced. In its revised form this volume continues to be an admirable exposition of modern gynecology.

A Hand-Book of Surgery.—For students and practitioners. By Frederic R. Griffith, M. D., Surgeon to the Bellevue Dispensary, New York City; Assistant Surgeon at the New York Polyclinic School and Hospital. 12mo. volume of 579 pages, containing 417 illustrations. Philadelphia, New York, London: W. B. Saunders & Company. 1904. Flexible leather, $2.00 net.

Dr. Griffith has given us a little work of great merit. It is a brief outline of the principles and practice of surgery, written as concisely as is possible with clearness. We are sure it will be valuable alike to the student and the practitioner, because the entire subject of surgery is covered, including all the specialties, as diseases of the eye, ear, nose and throat; genito-urinary diseases; diseases of women, etc. There are also articles on life insurance, rape, sexual perversions, microscopy, and on many other subjects of great importance to the practicing surgeon. There are 417 illustrations, selected for their clearness, accuracy, and general usefulness. We predict that Dr. Griffith's work will be to surgery what Dr. Stevens' manual is to medicine.

---

A Text-Book of Materia Medica.—Including laboratory exercises in the histologic and chemic examinations of drugs. For pharmaceutic and medical schools, and for home study. By Robert A. Hatcher, Ph. G., M. D., Instructor in Pharmacology in Cornell University Medical School of New York City; and Torald Sollmann, M. D., Assistant Professor in Pharmacology and Materia Medica in the Medical Department of the Western Reserve University of Cleveland. 12mo. volume of about 400 pages, illustrated. Philadelphia, New York, London: W. B. Saunders & Company. 1904. Flexible leather, $2.00 net.

Students of medicine, as well as pharmacy students, will undoubtedly welcome this work. The authors are teachers of much experience, and in this forelying work present a work on the subject of materia medica in an entirely new way, teaching by actual experimental demonstrations. Part I comprises a guide to the study of crude drugs, both official and unofficial; while in parts II and III the histologic and chemic examinations of drugs are considered in a scientific, clear and simple manner. All the histologic descriptions are supplemented by laboratory exercises of important drugs, so that the student becomes insensibly acquainted with their construction. Throughout the entire work general stress is laid on the recognition of adulterations. We can strongly recommend this work as reliable, practical, and excellent in every way.

---

Manual of Materia Medica and Pharmacy.—Specially designed for the use of practitioners and medical, pharmaceutical, dental, and veterinary students. By E. Stanton Muir, Ph. G., V. M. D., Instructor in Comparative Materia Medica and Pharmacy in the University of Pennsylvania. Third edition, revised and enlarged. Crown octavo, 192 pages, interleaved throughout. Bound in extra cloth, $2.00 net. F. A. Davis Company, Publishers, 1914-16 Cherry Street, Philadelphia, Pa.

This book is intended, says the author, to give, in as concise and clear a manner as possible, those points which are of value, without the lengthy detail usually found in text-books. Many new and some old drugs have been intentionally omitted, and only those are considered which are in daily use and of recognized therapeutic value. In other words, there has been a culling of the wheat from the chaff, for works on materia medica usually contain long accounts of drugs that are little known and seldom used. In this the author has been a benefactor.

---

Obstetrics and Gynecologic Nursing. By E. P. Davis, A. M., M. D., Jefferson Medical College, and Philadelphia Polyclinic. Second edition, revised. W. B. Saunders & Co., Philadelphia, New York and London. 1904. 387 pages. $1.75.

This book was especially prepared for use in the training schools of the Philadelphia and Jefferson Hospitals, the writer being an instructor in both those schools. It is, of course, adapted to other schools, and contains a pretty full course of instruction which will be found very useful to those who intend to nurse such cases.

---

Refraction and Motility of the Eye.—For students and practitioners. By Wm. Norwood Suter, M. D. 101 engravings and four colored plates. 380 pages. Cloth. Lea Bros & Co., Philadelphia and New York. Price, $2.00 net.

This work is simple enough for beginners in ophthalmology and yet complete enough to meet the requirements of more advanced students and practitioners. That is a recommendation. Most authors presuppose too much knowledge on the part of readers.

---

Lectures on Diseases of the Nervous System.—Second series. Blakiston's Son & Co., Philadelphia. Subjective Sensations of Light and Sound.—Abitrophy and other lectures. By Sir Wm. R. Gowers, M. D., F. R. C. P., F. R. S.

These lectures have been revised and partially rewritten by the author. He says, however, that "there is, indeed, a prospect that our ultimate conceptions may be thrown into the crucible, and recast by the influence of ardent thought, through the discoveries regarding radio-activity, and the possible nature of the elementary constituents of matter." The book, however, is up to date.

---

A Text-Book of Physiology. By Isaac Ott, A. M., M. D., Professor of Physiology in the Medico-Chirurgical College of Philadelphia. With 137 illustrations. Royal octavo, 563 pages. Bound in extra cloth. Price, $3.00 net. F. A. Davis Company, Publishers, 1914-16 Cherry Street, Philadelphia, Pa.

This book does not aim to be a treatise on the subject, but is an elementary work containing the chief facts of physiology which are necessary to a practitioner. Physiology being the basis of medicine, its understanding, says the author, is requisite to the study of pathology. Such a work will be appreciated by students and beginners, I am sure.

---

THE MEDICAL EPITOME SERIES—PEDIATRICS.—A manual for students and practitioners. By Henry Enos Tuley, A. B., M. D., Medical Department University of Kentucky. Series edited by V. C. Peterson, A. M., M. D., New York Polyclinic Medical School and Hospital. Illustrated with thirty-three engravings. Lea Brothers & Co., Philadelphia and New York. 1904. Cloth, boards, 250 pages. Price, $1.00.

This is intended to "aid the student in acquiring command of this important subject," says the author. It is a selection and condensation of accepted knowledge, as its name implies. The selections and authorities are Holt, Roche, Jacobi, Corlett, Nothnagel's Encyclopedia, Sachs, Ashby, Wright, Cotton and others.

---

RADIOTHERAPY, PHOTOTHERAPY AND HIGH FREQUENCY CURRENTS. —The medical and surgical applications of radiology in diagnosis and treatment. By Charles Warrenne Allen, M. D., Professor of Dermatology in the New York Post-Graduate Medical School. Octavo, 618 pages, 131 engravings and twenty-seven plates. Cloth, $4.50 net. Lea Brothers & Co., Publishers, Philadelphia and New York.

Recent discoveries in radiant energy have developed a new and important system of therapy. In fact, such positive results have already been achieved in maladies which were hitherto considered intractable, as to warrant the recognition of radiotherapy as a very efficient addition to the resources of the profession. Dr. Allen's work is peculiarly opportune. It is based upon practical experience as well as upon a careful review of the great mass of literature on the subject coming from almost all quarters of the globe. Naturally, in a science so new, much faulty obesrvation has been encountered, and in this volume no effort has been spared to eliminate the errors and to present the subject correctly and abreast of its position today. Ample information is given upon the physical as well as the technical side, to equip the reader for the selection and management of appliances. The object of the work is always practical, and it has been the earnest endeavor of the author to enable his readers to secure for their patients prompt and permanent benefit. Accordingly, much attention is given to questions of diagnosis and treatment, and, in as much as such powerful forces as are treated of in this volume may do harm if properly applied, cautionary directions are carefully given and exact instructions for the determination and measurement of dosage.

The author has by no means lost sight of the demands of undergraduate students, and his long teaching experience has enabled him to produce a work which is admirably adapted to teaching purposes.

